# Experimental Nonevidence of Fragile-to-Strong Crossover

**DOI:** 10.1021/acsmaterialslett.5c01178

**Published:** 2025-12-02

**Authors:** Petr Koštál, Jaroslav Barták, Michaela Včeláková, Stanislav Slang, Torsten Wieduwilt, Markus A. Schmidt, Jiří Málek

**Affiliations:** † Department of Inorganic Technology, 48252 University of Pardubice, Doubravice 41, 53210 Pardubice, Czech Republic; ‡ Department of Physical Chemistry, University of Pardubice, Studentská 573, 53210 Pardubice, Czech Republic; § Center of Materials and Nanotechnologies, University of Pardubice, Nam. Cs. Legii 565, 53002 Pardubice, Czech Republic; ∥ 40096 Leibniz Institute of Photonic Technology, Albert-Einstein-Str. 9, 07745 Jena, Germany; ⊥ Abbe Center of Photonics and Faculty of Physics, Friedrich-Schiller-University Jena, Max-Wien-Platz 1, 07743 Jena, Germany; # Otto Schott Institute of Material Research, Friedrich-Schiller-University Jena, Fraunhoferstr. 6, 07743 Jena, Germany

## Abstract

This study explores the viscosity behavior of the Ge–Se chalcogenide glass-forming system. Four compositions containing 5, 10, 15, and 20 at. % germanium were examined. Viscosity measurements were performed over a broad range, spanning approximately 13 orders of magnitude, by combining the pressure-assisted melt filling technique with penetration and parallel-plate viscometry. The results demonstrate that no fragile-to-strong crossover occurs in any of the studied compositions.

## Graphical abstract

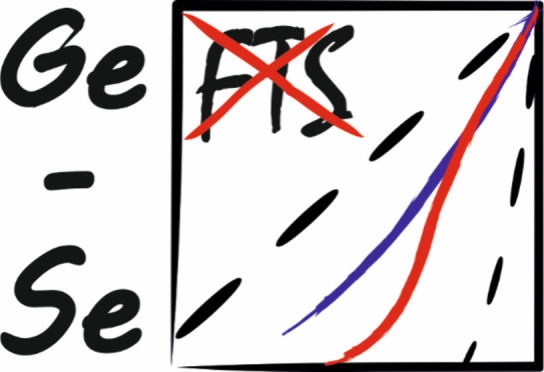

The fragile-to-strong crossover or transition (abbreviated as FTS or F–S) is a widely discussed phenomenon in materials science, particularly in the study of glass-forming systems. This phenomenon is most commonly visualized using the so-called Angell plot^1^ (Figure 1), which provides a normalized representation of the temperature dependence of viscosity.

**Figure 1:**
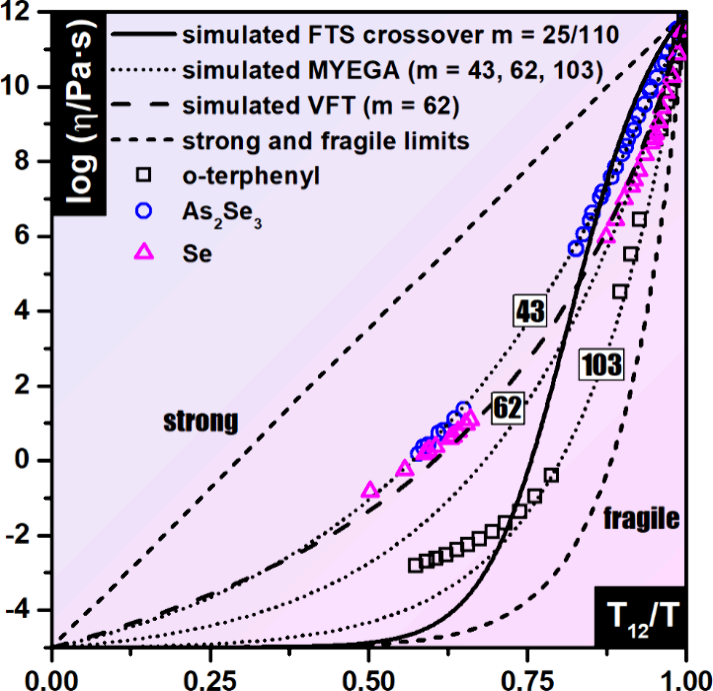
Angell plot showing the logarithmic dependence of viscosity on the reduced temperature *T*_12_/*T*. Experimental viscosity data for *o*-terphenyl,^2,3^ As_2_Se_3_,^6,7^ and Se^6,8^ are shown alongside simulated viscosity curves calculated using the MYEGA and VFT models.

The conceptual foundation of Angell plot was initially introduced by Laughlin and Uhlmann^2^ and was later extensively developed by C. A. Angell throughout his distinguished career. In the standard form, the Angell plot displays the logarithm of dynamic viscosity as a function of reduced temperature *T*_12_/*T*, where *T*_12_ denotes the temperature at which the dynamic viscosity reaches 10^12^ Pa·s. The *T*_12_ temperature, commonly referred to as the viscosity glass transition temperature, is widely accepted by convention. The point was determined by definition because no anomaly is observable at the glass transition on viscosity curve.

In the Angell plot, the behavior of glass-formers is characterized by the so-called fragility parameter, which captures the deviation from Arrhenius-like behavior. Systems exhibiting Arrhenius behavior of viscosity over a wide temperature range are classified as strong and appear as straight lines on the Angell plot. These systems are characterized by low fragility values, with amorphous GeO_2_ being a classic example.^1^ In contrast, fragile glass-formers exhibit pronounced non-Arrhenius behavior, corresponding to high fragility parameters. Organic compounds such as 1,2-diphenylbenzene (commonly known as *o*-terphenyl or OTP)^2,3^ and 1,3-bis­(1-naphthyl)-5-(2-naphthyl)­benzene (TNB)^4^ are typical representatives of this group. In addition to dynamic viscosity, the Angell plot can also be constructed using relaxation time, as it is generally proportional to viscosity through an approximately temperature-independent instantaneous (high-frequency) shear modulus.^5^ This approach forms the basis for the kinetic classification of fragility.

A complementary perspective was introduced by Kauzmann,^9^ leading to the concept of thermodynamic fragility. This classification is based on the rate of excess entropy loss upon cooling from the melting point. Excess entropy is defined as the difference between the entropies of the liquid and corresponding crystalline states. In Kauzmann’s approach, reduced temperature *T*/*T*_m_ is used to plot excess entropy normalized by melting entropy. A related representation compares the excess entropy at the glass transition divided by the total excess entropy, plotted against *T*_g_/*T*, offering a thermodynamic analog to the kinetic fragility classification. While theoretical frameworks such as the energy landscape theory^10^ suggest that kinetic and thermodynamic fragilities need not coincide, some studies have reported good agreement,^11^ whereas others have observed notable discrepancies.^12^ In summary, although parallels between the two classifications are often observed, significant deviations can arise for specific materials, highlighting the complex nature of glass-formers’ behavior.

The fragile-to-strong (FTS) crossover, mentioned earlier in the text, is typically defined as the manifestation of dual fragility behavior in a given material, preferably within the framework of a single fragility classification method. In essence, a glass-forming system exhibiting FTS crossover behaves as a strong liquid in the vicinity of the glass transition but displays significantly more fragile characteristics at higher temperatures in the melt. This phenomenon is most commonly associated with the kinetic classification of fragility. An illustrative example of FTS behavior is presented in Figure 1, showing a simulated viscosity curve generated using the generalized MYEGA model^13^ with steepness index values of 25 and 110 in the low- and high-temperature regions, respectively. The steepness index *m*, which is the most widely adopted quantitative measure of fragility, is defined as^14^

1 $$m=\underset{T\to T_{12}}{\lim}\frac{d\;\log\left(\eta \;\mathrm{or}\;\tau \right)}{d\left(T_{12}/T\right)}$$

where η stands for the dynamic viscosity and τ stands for the relaxation time. The steepness index thus represents the slope of the viscosity (or relaxation time) plotted in Angell coordinates in the proximity to the glass transition. Importantly, this definition strictly applies to the temperature region close to *T*_12_, and its use outside this range can lead to misinterpretation. While the steepness index is sometimes reported for data far from *T*_12_. Such practice neglects this limitation and should be approached with caution. The use of a single fragility parameter *m* to characterize an entire viscosity curve is the simplification of real glass-formers viscosity behavior.

Even the simplest equations used to describe the viscosity of nonstrong glass-formers involve three parameters. In the commonly used reformulated expression introduced by Mauro et al.,^15^ these parameters include the steepness index, the viscosity glass transition temperature, and the viscosity at infinite temperature, usually denoted as log η_0_ or log η_∞_. While the Angell plot assumes a universal value of −5 for log *η*_∞_, this value remains experimentally unverifiable due to the significant extrapolation required from available viscosity data. Therefore, although the existence of a single viscosity value at infinite temperature is plausible, it remains hypothetical. Furthermore, the type of the chosen viscosity equation significantly influences the predicted curve. The difference is naturally more evident in the melt region due to the significant departure from the fixed viscosity points (for Angell plot 10^12^ and 10^–5^ Pa·s). For example, in Figure 1, two theoretical viscosity curves with the same steepness index value (62) are shown using the Vogel–Fulcher–Tammann (VFT)^16–18^ and Mauro–Yue–Ellison–Gupta–Allan (MYEGA)^15^ equations. Even with identical parameters (*m*, *T*_12_, log η_0_), the curves differ by approximately 1.4 orders of magnitude at a reduced temperature of 0.5. Figure 1 also presents theoretical MYEGA curves corresponding to specific steepness index values determined from experimental viscosity data for As_2_Se_3_,^6,7^ Se,^6,8^ and OTP.^2,3^ The values 43 (As_2_Se_3_), 62 (Se), and 103 (OTP) represent the best fits to the respective viscosity data sets, obtained without fixing any parameters, whereas the theoretical curves were constrained by setting log η_0_ to −5. It is evident that the viscosity behavior of As_2_Se_3_ is well described by a theoretical MYEGA curve using a single steepness index value. In contrast, selenium exhibits behavior that has been termed “fragility disparity”.^19^ Systems exhibiting this behavior are characterized by distinct fragility parameters determined independently for the melt and undercooled melt regions. Fragility disparity does not reflect any transition; rather, it arises from the complex nature of viscosity behavior and the simplifications inherent in the determination and interpretation of fragility. On the other hand, the FTS crossover discussed in this work represents a liquid–liquid transition with an as yet unidentified origin.

The FTS crossover was first identified by Ito et al.^20^ based on calorimetric measurements. The work reported that water behaves kinetically as a strong liquid near the glass transition but exhibits thermodynamically fragile behavior in the liquid. Subsequent studies described the phenomenon for other systems. For example, Lucas^21^ identified FTS crossover in ZnCl_2_ based on calorimetric determination of kinetic fragility near *T*_g_ and viscosity data in the melt region. Similarly, Zhang et al.^13^ reported FTS behavior in metallic glasses, inferred from indirect viscosity estimates in the undercooled region^22^ and oscillating cup measurements in the melt.^23^ More direct approaches using rotational and beam-bending viscometry also confirmed FTS crossover in metallic glass-formers.^24,25^ Beyond metallic systems, FTS behavior has also been frequently reported for chalcogenide materials. Ultrafast scanning calorimetry revealed FTS crossover in chalcogenide phase-change materials (PCMs),^26,27^ where it is regarded as a potentially critical property for their technological applicability.^26,28^ A comprehensive review by Lucas^29^ further explores the FTS crossover. A significant part of Lucas’ work^29^ deals with the chalcogenide materials and the existence of the crossover in Ge_15_Te_85_ and compositions from Ge–Se system. Notably, the FTS crossover was detected not only through variations in fragility but also via anomalies in heat capacity and thermal expansion behavior.^29^

The detection of the FTS crossover is often based on indirect evidence. This is primarily because systems that exhibit such transitions are typically poor glass-formers, which prevents the preparation of bulk samples and makes it difficult to determine their viscosities in the glassy and undercooled melt regions using conventional techniques. Nevertheless, the FTS crossover has also been reported in several sufficiently good glass-forming systems. In addition to the previously mentioned studies on metallic glass-formers,^24,25^ compositions from the chalcogenide Ge–Se system are frequently cited in connection with FTS crossover observations. These reports are supported by direct viscosity measurements: in the undercooled melt region by Nemilov,^30^ and in the melt region by Laugier et al.,^31^ and Glazov and Situlina.^32^ The melt viscosity data in the latter two studies were obtained using the oscillating cup (or oscillating vessel) method,^33^ also known historically as the Meyer–Shvidkovski method.^34,35^ This technique involves measuring the damping of oscillations of a crucible filled with the liquid sample. Typically, the crucible is suspended on a torsion wire and set into torsional oscillation about a vertical axis. The damping of oscillation, which is caused by viscous energy dissipation in the liquid, is then analyzed. The oscillating cup method is widely used for high-temperature viscosity measurements of molten metals and alloys,^36,37^ and it has historically been the most frequently applied method for chalcogenide melts.^38^ It offers several advantages, including the ability to measure highly volatile and chemically aggressive melts due to the possibility of sealing the sample within a crucible or ampule. Additionally, it requires only a small sample volume and enables relatively easy and precise measurement of oscillation damping.^39^ However, despite its experimental convenience, the method and its theoretical foundation are complex. Determining viscosity requires solving a second-order differential equation and the Navier–Stokes equations describing fluid motion in the crucible.^39^ The derivation of working equations has been published in several studies,^40^ with widely used approaches proposed by Roscoe^23^ and Shvidkovski.^35^ Nevertheless, it has been pointed out that these mathematical treatments may inadequately account for the wetting behavior of the liquid on the crucible walls,^36^ potentially resulting in inaccurate viscosity values.

This issue was also raised in the study by Zhu et al.,^41^ who compared viscosity data for Ge_15_Se_85_ and Ge_20_Se_80_ compositions obtained via capillary and parallel-plate methods with values reported by Laugier et al.,^31^ and Glazov and Situlina.^32^ Their results revealed discrepancies of nearly 1 order of magnitude, leading them to challenge the validity of FTS crossover observations in these materials. Lucas,^42^ however, contested this conclusion, suggesting that the discrepancies could be attributed to compositional shifts caused by vapor losses under high-temperature conditions during Zhu et al.’s measurements.

In light of these conflicting reports, we performed our own viscosity measurements on four compositions from the Ge–Se system: Ge_5_Se_95_, Ge_10_Se_90_, Ge_15_Se_85_, and Ge_20_Se_80_. The measurements cover a wide temperature range, spanning approximately 13 orders of magnitude in viscosity. A combination of three experimental techniques was employed: thermomechanical analysis (TMA),^43^ using both parallel-plate^44^ and penetration viscometry,^45–49^ was used for the undercooled melt and glassy regions, while the pressure-assisted melt filling technique (PAMFT)^6,50^ was applied in the melt region. Details of the measurements and data (Tables S2 and S3) are provided in the Supporting Information. Our viscosity data in the undercooled melt and glassy states show good agreement with previously published values for Ge_10_Se_90_ by Pustková et al.^51^ and for Ge_10_Se_90_ and Ge_20_Se_80_ by Gueguen et al.^52^ The earlier data by Nemilov^30^ show minor deviations from ours in some compositions and temperature intervals, though overall consistency remains acceptable (see Figure S1 in the Supporting Information). Viscosity data in the melt region also align well with those reported by Zhu et al.^41^ for Ge_15_Se_85_ and Ge_20_Se_80_. Additionally, our data for Ge_10_Se_90_ are in good agreement with those of Perron et al.^53^ In contrast, the viscosity values reported by Laugier et al.,^31^ measured using the oscillating cup method, deviate significantly from ours and other sources. For Ge contents of 10, 15, and 20 at. %, the discrepancies reach up to 1 order of magnitude. For the 4.5 at. % Ge composition reported by Laugier et al.^31^ (compared with our Ge_5_Se_95_), the deviation is smaller, less than half an order of magnitude, though not fully explainable by the slight compositional difference. Lucas^42^ also noted that for pure selenium, the oscillating cup method yields viscosity values consistent with classical techniques, supporting Zhu et al.’s conclusion^41^ that the method becomes unreliable as germanium content increases. To address Lucas’^42^ concerns about compositional changes during capillary measurements, we performed energy-dispersive X-ray spectroscopy on our postmeasurement samples. The analysis confirmed that no compositional changes occurred (see Supporting Information). This strongly supports the accuracy of our data and casts further doubt on the results reported by Glazov and Situlina^32^ for Ge_10_Se_90_ and Ge_20_Se_80_. Although those data were obtained in different temperature ranges and at lower viscosities, they too exhibit noticeable discrepancies.

When plotted in Angell coordinates (Figure 2), the inconsistencies between our data and the oscillating cup results from Laugier et al.,^31^ and Glazov and Situlina^32^ become clearly evident. This visualization reinforces the assertion by Zhu et al.^41^ that the previously reported FTS crossover in the Ge–Se system likely arose from inaccuracies in oscillating cup measurements. Our data for all studied compositions can be described using viscosity curves with a single steepness index, clearly indicating the absence of an FTS crossover in these Ge–Se glass-formers.

**Figure 2:**
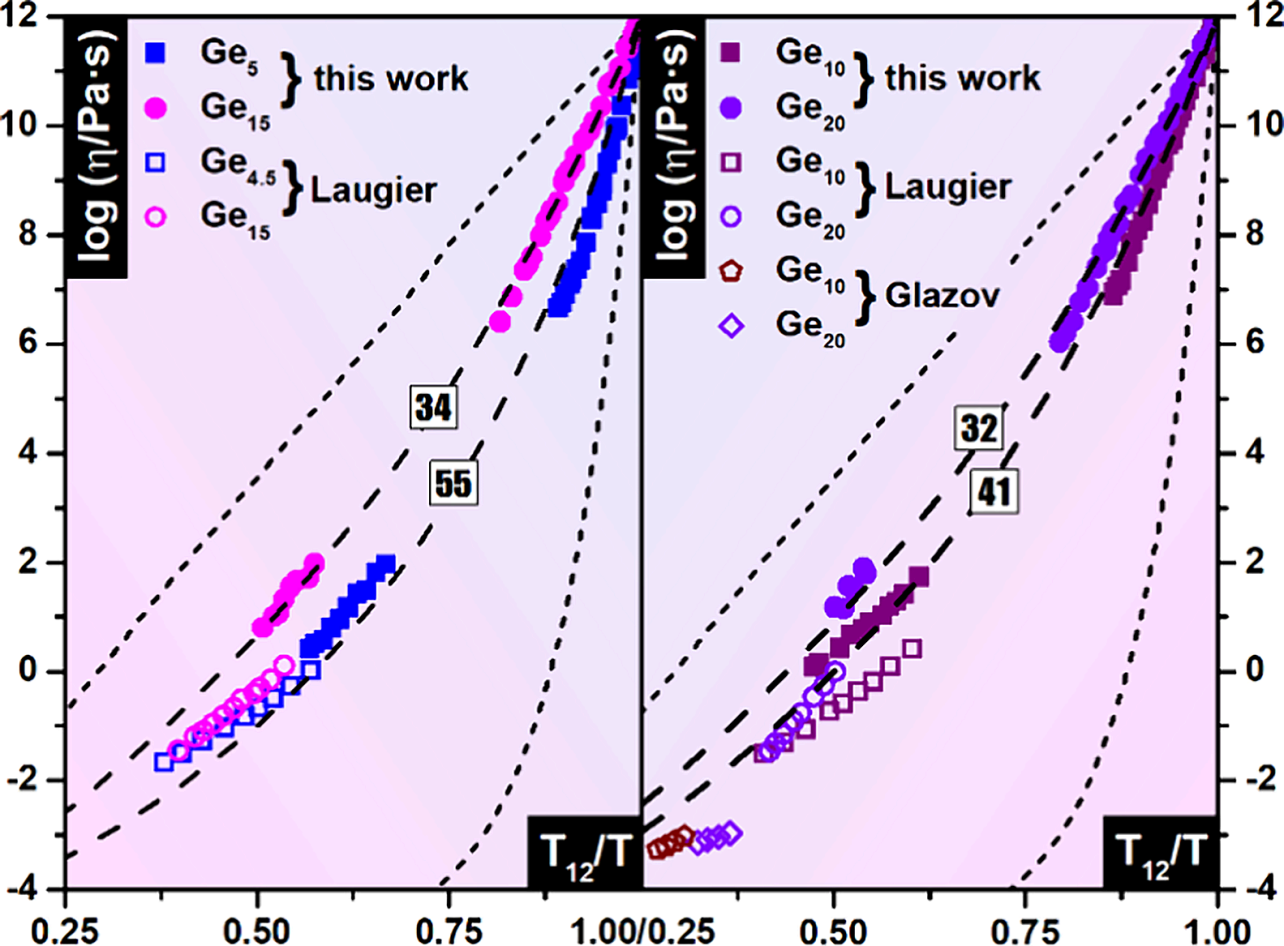
Experimental viscosity data for Ge_*x*_Se_100–*x*_ (*x* = 5, 10, 15, and 20) plotted in Angell coordinates. Data obtained in this work are shown alongside previously published results by Laugier et al.,^31^ and Glazov and Situlina.^32^ Theoretical VFT model curves corresponding to the indicated steepness index values are also included for comparison.

It is evident from Figure 2 that the theoretical VFT viscosity curves do not perfectly fit the experimental data. This discrepancy arises from the limitation previously discussed: the theoretical curves represent the VFT model with a fixed value of viscosity at infinite temperature, log η_0_ = – 5. This universal value was originally proposed by Angell.^1^ However, fits to experimental data frequently deviate from this assumption.

The inconsistencies between our data and the oscillating cup data are clearly visible based solely on the experimental results, without the need for any fitting. Figure 3 presents the classical Arrhenius representation of the viscosity data obtained in this study, together with the originally kinematic viscosity data reported by Laugier et al.^31^ and Glazov and Situlina.^32^ The discrepancies that previously led to the false identification of an FTS crossover in this glass-forming system are readily apparent.

**Figure 3:**
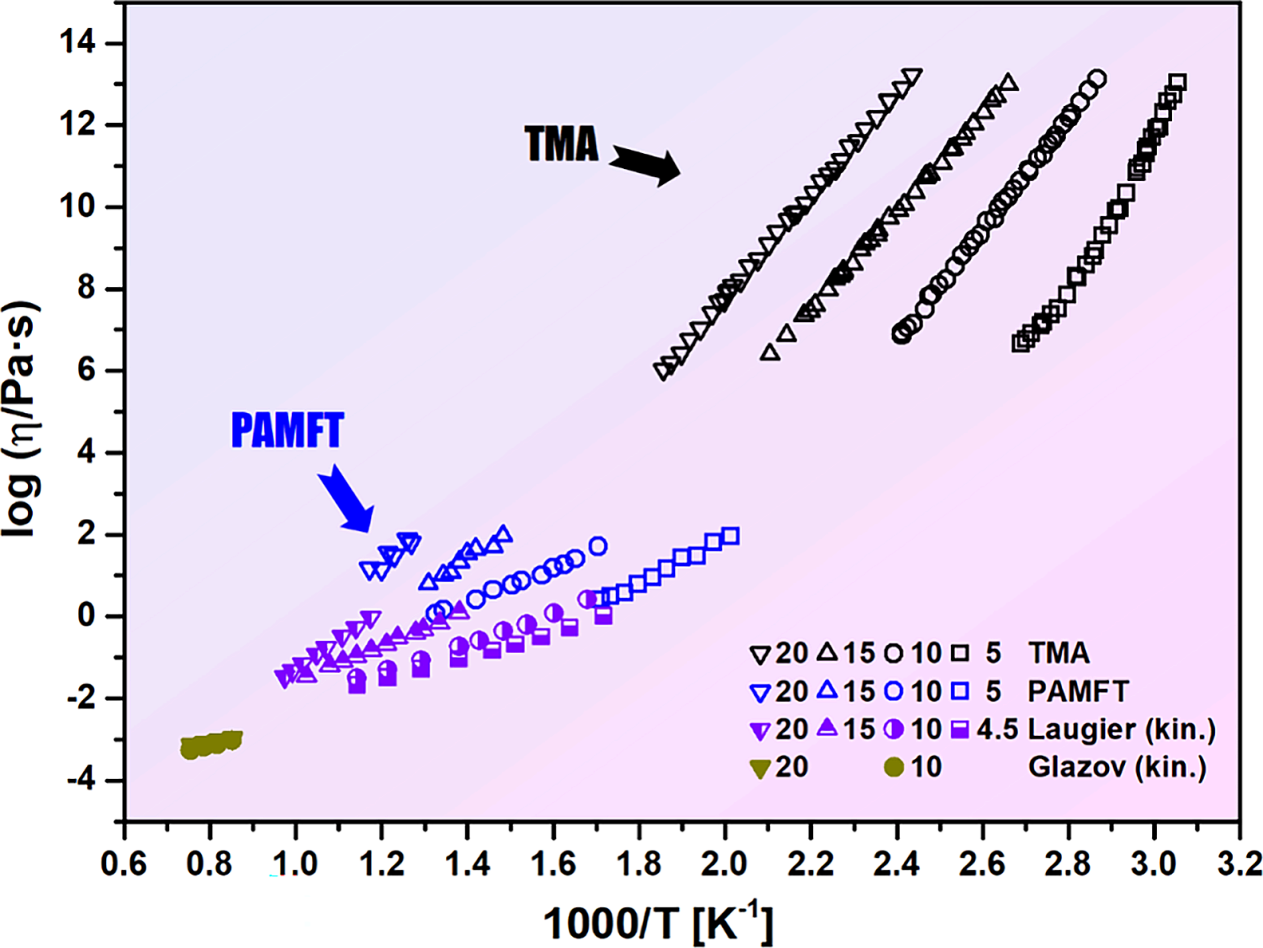
Experimental viscosity data for Ge_*x*_Se_100–*x*_ (*x* = 5, 10, 15, and 20) plotted in the Arrhenius coordinates. Data obtained in this work are shown alongside previously published results by Laugier et al.^31^ and Glazov and Situlina.^32^

All literature data are summarized in Figure S1 of the Supporting Information. The experimental data presented in this work were also fitted using several viscosity models without constraining any parameters, namely the Vogel–Fulcher–Tammann (VFT),^16–18^ the Mauro–Yue–Ellison–Gupta–Allan (MYEGA),^15^ and the Krausser–Samwer–Zaccone (KSZ)^54^ equations. The fitting parameters (*m*, *T*_12_, log η_0_) are summarized in Table S4 of the Supporting Information. The classical form of the KSZ model was reformulated into a version expressed in terms of these parameters (eq 2), allowing for a more direct comparison between the different models.

2 $$\log\eta =\log\eta_{0}+\left(12-\log\eta_{0}\right)\cdot \frac{T_{12}}{T}\cdot \exp\left[\left(\frac{m}{12-\log\eta_{0}}-1\right)\cdot \left(1-\frac{T}{T_{12}}\right)\right]$$

The original parameter values of each equation can be readily obtained by fitting the experimental data summarized in Tables S2 and S3 in the Supporting Information. This is particularly important when considering the physical meaning of the parameters and their broader implications for material behavior.^54–57^

## Conclusion

The experimental data presented in this work demonstrate that the previously reported fragile-to-strong (FTS) crossover in the Ge–Se glass-forming system, based on viscosity measurements, was likely the result of inaccuracies introduced by the oscillating viscometry method. Nevertheless, several important remarks should be made in connection with our study:

1. This finding does not discredit all data obtained via oscillating viscometry. For instance, viscosity data for pure selenium measured using this method are in good agreement with results from other experimental techniques. However, viscosities determined by oscillating viscometry should be treated with caution, as they may be affected by additional experimental uncertainties, likely related to the wetting behavior of the sample against the crucible wall.
2. This work does not challenge the general existence of the FTS crossover phenomenon.
3. Nor does it invalidate the anomalies in heat capacity and thermal expansion summarized in the study by Lucas^29^ and others. However, if FTS crossover does exist within the Ge–Se glass-forming system, it is not manifested in the temperature dependence of viscosity. The viscosity behavior of the four studied compositions is representative and consistent with trends observed in the majority of glass-forming systems.

## Supplementary Material



## Data Availability

The data that support the findings of this study are openly available in ZENODO at https://zenodo.org/, reference number 10.5281/zenodo.15790542.

## Notes

Synthesis method, sample preparation, verification of composition, details of viscosity measurement, coefficients of linear thermal expansion, determined viscosity values, comparison with literature data, VFT, MYEGA and KSZ parameters of fits through experimental data (PDF)

## Abbreviations

FTS: fragile-to-strong crossover
KSZ: Krausser–Samwer–Zaccone model
MYEGA: Mauro–Yue–Ellison–Gupta–Allan model
OTP: Twelve diphenylbenzene
PAMFT: pressure-assisted melt filling technique
PCM: phase change materials
TNB: 13-bis­(1-naphthyl)-5-(2-naphthyl)­benzene
VFT: Vogel–Fulcher–Tammann model
