# Supplementary material for: Experimental Nonevidence of Fragile-to-Strong Crossover
Source: ACS Mater Lett. 2025 Dec 2;8(1):116–22. doi: 10.1021/acsmaterialslett.5c01178 (PMC12776574; doi:10.1021/acsmaterialslett.5c01178)
Supplement: Supplementary file 1 [file tz5c01178_si_001.pdf]

Supporting Information

Experimental nonevidence of fragile-to-strong crossover

Petr Koštál[1,*], Jaroslav Barták[2], Michaela Včeláková[1], Stanislav Slang[3], Torsten Wieduwilt[4], Markus A. Schmidt[4,5,6], Jiří Málek[2]

[1]Department of Inorganic Technology, University of Pardubice, Doubravice 41, 53210 Pardubice, Czech Republic

[2]Department of Physical Chemistry, University of Pardubice, Studentská 573, 53210 Pardubice, Czech Republic

[3]Center of Materials and Nanotechnologies, University of Pardubice, Nam. Cs. Legii 565, 53002 Pardubice, Czech Republic

[4]Leibniz Institute of Photonic Technology, Albert-Einstein-Str. 9, 07745 Jena, Germany

[5]Abbe Center of Photonics and Faculty of Physics, Friedrich-Schiller-University Jena, Max-Wien-Platz 1, 07743 Jena, Germany

[6]Otto Schott Institute of Material Research, Friedrich-Schiller-University Jena, Fraunhoferstr. 6, 07743 Jena, Germany

Experimental Section

Sample preparation

Glassy samples of Ge_xSe_{100-x} (where x = 5, 10, 15, and 20) were synthesized via the classical melt-quenching method. The appropriate amounts of high-purity (5N) elemental germanium and selenium were weighed and sealed under vacuum (~10^{-3} Pa) in silica ampoules. The synthesis was realized in a rocking furnace. The ampoules were initially heated to 1073 K at a rate of 10 K·min^{-1} and held for 20 hours, followed by cooling to 1023 K. Subsequently, the temperature was increased to 1323 K at a slow heating rate of 0.1 K·min^{-1} and maintained isothermally for at least 12 hours. The ampoules were then cooled to 1023 K at the same rate and finally quenched in iced water. The amorphous nature of the resulting samples was confirmed by X-ray diffraction (XRD) using a Rigaku MiniFlex 600 diffractometer (Bragg-Brentano 2θ–2θ geometry, CuKα radiation, λ = 1.5418 Å, U = 400 kV, I = 15 mA).

Samples intended for thermomechanical analysis (TMA) were cut from the bulk glass using a low-speed diamond saw (IsoMet, Buehler). Thin glass plates (~6 × 6 × 2.5 mm^3) or cylindrical samples (6 mm in diameter, 2.5 mm thick) were prepared for penetration and parallel-plate measurements, respectively.

For pressure-assisted melt filling technique (PAMFT), fibers with diameters between 60 and 80 μm were used. These were fabricated by melting ~2 g of the bulk glass in an open silica ampoule in an inert nitrogen atmosphere, heated approximately 30 °C above the liquidus temperature. Fine glass fibers were drawn by rapidly pulling a silica rod immersed in the melt. Selected fibers were subsequently cut and used in filling experiments. Possible compositional deviations between fibers and the initial bulk material were examined using an energy-dispersive X-ray spectrometer (EDS; Aztec X-Mac 20, Oxford Instruments) integrated with a scanning electron microscope (SEM; LYRA 3, Tescan, Czech Republic). EDS measurements were performed at an acceleration voltage of 20 kV. The compositions of the fibers agreed with those of the parent bulk glasses within ±0.5 at.%. The same instrumentation was used to analyze the material that had been filled into the capillaries during the PAMFT measurements

Viscosity measurement

Viscosity measurements were realized using three complementary experimental techniques. Penetration and parallel-plate methods were applied for the high-viscosity range (approximately from 10^6 to 10^{13} Pa·s), while PAMFT was employed for measuring low viscosities (approximately from $10^{0.5}$ to 10^2 Pa·s) in the melt.

Thermomechanical analysis was performed using two instruments: TMA CX03 (RMI, Czech Republic) and TMA PT 1600 (Linseis, Germany). These instruments measure changes in sample height with high precision, using a differential capacitance displacement detector (CX03) and a linear variable differential transformer (PT 1600). Temperature calibration was performed using the melting points of pure metals (Ga, In, Sn, Pb, Zn, Al). Detailed descriptions of instrument calibration and measurement accuracy are available in our previous work[1].

The penetration technique, originally introduced by Cox[2] more than eighty years ago[2], determines viscosity by monitoring the penetration depth or rate of an indenter under constant load. Different shapes of indenters can be used. Nevertheless, hemispherical and cylindrical indenters are utilized most frequently. Both these shapes of indenters were used in this study. Specifically, corundum hemispherical indenter (diameter 3.98 mm) and stainless-steel cylindrical indenters (diameters 0.5 and 1 mm) were employed. The viscosity of sample penetrates by hemispherical indenter is calculated by[3, 4]:

$$\eta = \frac{9}{32\sqrt{2r}}\frac{Ft}{h^{3/2}}, \quad (1)$$

where F stands for the applied force, t stands for the time of penetration, h stands for the penetration depth, and r stands for the radius of the hemisphere. The theoretical description of the indentation process performed by cylindrical indenter was published in the works of Yang and Li[5, 6]. The working equation which calculates viscosity determined by cylindrical indenter follows (the meanings of symbols are same as in the previous equation)

$$\eta = \frac{F}{8r(dh/dt)}. \quad (2)$$

The penetration method is effective for viscosities in the range 10^8 to 10^{13} Pa·s. Viscosities above this range are impractical to measure due to excessive equilibration times, while lower viscosities result in rapid penetration, even under low loads (~10 mN), limiting accuracy. To address this, the parallel-plate method was used to measure viscosities below 10^8 Pa·s. This technique involves compressing a cylindrical sample between two parallel plates under constant load and monitoring its deformation. The viscosity is calculated using[7]:

$$\eta = \frac{2\pi F d^5}{3V(dd/dt)(2\pi d^3 + V)}, \quad (3)$$

where d stands for the sample height, V stands for its volume, and t and F have the same meanings as in the previous equations (the time of deformation and the applied force). Knowledge of the sample volume at the measurement temperature is required for viscosity calculations based on Eq. 3. However, the influence of temperature-induced volume changes on the resulting viscosity values is relatively minor. Therefore, an estimated volume at the measurement temperature is generally sufficient. The volume can be calculated from room-temperature dimensions using the coefficient of thermal expansion (CTE). In this study, the CTE values were derived from experimentally determined coefficients of linear thermal expansion (CLTE), assuming isotropic behavior of the glass samples. The CLTEs were measured using thermomechanical analysis (TMA) in accordance with ASTM standards[8, 9].

Viscosity determination in the high-viscosity region requires substantial isothermal holding times. In this study, values near 10^{13} Pa·s required isotherms exceeding 100 hours, while those at lower viscosities (measured by the parallel-plate method) required ~30 minutes. All data points presented in the Results section were obtained under isothermal conditions to minimize thermal gradient-related errors. The parallel-plate measurements could be realized in a non-isothermal regime. Nevertheless, this procedure can be possibly influenced by higher experimental error connected with temperature lag or lead occurrence (for more details see ASTM standard C 1351 M[10] or our previous work[11]). The precision of the viscosity measurements is ±0.1 (log scale), and the overall accuracy of statistically best fit through experimental data is ± 0.05 (log scale), based on NBS standard measurements[1]. Temperature accuracy was maintained within ±0.5 K.

The PAMFT method is, in principle, capillary viscometry. Glass fibers (60–80 µm in diameter, 1–3 mm in length) were inserted into auxiliary capillaries (ID: 80 µm; OD: 200 µm), which were spliced onto filling capillaries (ID: 3.46, 4.26, or 5.8 µm). Capillary selection was based on expected viscosity, filling length, and time. To minimize error, fibers were pushed to the junction to reduce free space. The auxiliary capillary was then attached to the pressure system, spliced capillaries were purged with pure argon (20 min) and then placed on a horizontal hot plate. Pressurized argon (5–40 bar) was used to drive the molten material into the narrower filling capillary. All capillaries used were fabricated from the same type of silicate glass. The thermal uniformity of the hot plate was monitored using two thermocouples positioned along its length to ensure stable and homogeneous temperature conditions. At specified time intervals, the capillaries were removed from the hot plate and depressurized. The filled length of the sample within the filling capillary was measured using an optical microscope. To continue the experiment, the capillaries were reinserted on the hot plate under the same temperature and pressure conditions, and the filling process was resumed. This procedure was repeated several times to obtain the time-dependent evolution of the filled length. The viscosity was calculated from the linear dependence of filled length squared versus time, using the simplified equation[12]:

$$L^2 \approx \left(\frac{p \cdot R^2}{4\eta}\right) \cdot t, \quad (4)$$

where L stands for the length of the filled sample, R stands for the radius of the filling capillary, p stands for the applied argon pressure, and t stands for the filling time. Eq. 4 represents a simplified form of a more comprehensive model that also includes terms accounting for surface tension and the contact angle between the melt and the silica glass. The validity of this simplification under the experimental conditions has been tested and discussed in detail in our previous work[12]. The error analysis expected errors in viscosity up to ±0.05 (log scale) and up to ±1 K in temperature.

Results Section

Determination of CLTE

The coefficients of linear thermal expansion (CLTE, denoted as α) were determined using thermomechanical analysis (TMA) under a controlled heating rate of 2 K·min^{-1}, and the results are summarized in Table S1. The measurement accuracy, validated using a platinum standard, was within 5%. Above the glass transition temperature T_g, the values are associated with increased experimental uncertainty due to the onset of viscous flow, which caused deformation of the samples before equilibrium CLTE values could be fully established. This limitation arises from the one-dimensional nature of the measurement method. Nevertheless, the accuracy is sufficient for the purpose of viscosity determination using the parallel-plate method, which was the primary motivation for performing the CLTE measurements. Glass transition temperatures were determined during heating at the mentioned rate (2 K·min^{-1}). Prior to these measurements, the thermal history of each sample was standardized by holding them isothermally for 20 minutes at a temperature approximately 15 K above T_g, followed by cooling to room temperature at 2 K·min^{-1}. The obtained CLTE values were also utilized to calculate the temperature dependence of density, which is necessary for converting literature data on kinematic viscosity into dynamic viscosity. This was specifically applied to data originally reported by Laugier et al.[13], and Glazov and Situlina[14]. The CLTE values reported in this study are consistent with those published by Avetikyan and Baidakov[15] for Ge contents of 5, 10, and 15 at.%. Although the value for 15 at.% Ge was not explicitly listed in that work[15], it was obtained here via interpolation of adjacent compositions. Avetikyan and Baidakov[15] also reported densities at room temperature for mentioned compositions. For the $Ge_{20}Se_{80}$ composition, the density values were taken from the work of Ruska and Thurn[16]. The resulting differences between the densities calculated using our CLTEs and values published in the mentioned works[15, 16] are within 5% at the temperatures corresponding to the viscosities reported by Laugier et al.[13], and Glazov and Situlina[14].

Table S1. Coefficients of linear thermal expansion

Composition	CLTE glass (α_g) $\cdot 10^6$ [K^{-1}]	CLTE melt (α_m) $\cdot 10^6$ [K^{-1}]	T_g [K]
**$Ge_{05}Se_{95}$**	46.0 ± 0.34	123 ± 5	331
**$Ge_{10}Se_{90}$**	41.0 ± 0.12	112 ± 5	357
**$Ge_{15}Se_{85}$**	33.2 ± 0.23	99 ± 5	380
**$Ge_{20}Se_{80}$**	27.6 ± 0.08	94 ± 3	424

Note: The mentioned standard deviations were calculated from at least 10 measurements, representing the precision of CLTE determination.

Viscosity measurement

The viscosities of Ge_xSe_{100-x} (x = 5, 10, 15, and 20) determined by thermomechanical analysis (TMA) techniques (penetration and parallel-plate methods) as well as by the pressure-assisted melt filling technique (PAMFT), are summarized in Tables S2 and S3, respectively. These data are also plotted in Figure S1, where they are compared with previously published experimental results by Perron et al.[17], Pustková et al.[18], Gueguen et al.[19], Zhu et al.[20], and Nemilov[21]. Data reported by Webber and Savage[22] for $Ge_{20}Se_{80}$ are not included in the figure due to their low precision, although they are partially consistent with our measurements. Data from Senapati and Varshneya[23] were omitted from the comparison as they are systematically shifted relative to other results. Viscosity data originally reported as kinematic viscosities by Laugier et al.[13], and Glazov and Situlina[14], obtained via the oscillating cup (Meyer-Shvidkovskii) method[24, 25], were converted to dynamic viscosities using our CLTE values summarized in Table S1 and densities determined at room temperature by Avetikyan and Baidakov work[15]. The curves shown in Figure S1 represent fits to our experimental data only. The Vogel–Fulcher–Tammann (VFT)[26-28], Mauro–Yue–

Ellison–Gupta–Allan (MYEGA)[29], and Krausser–Samwer–Zaccone (KSZ)[30] models were employed to describe the temperature dependence of viscosity. The resulting fitting parameters, together with the coefficient of determination (R^2) and the Akaike information criterion (AIC), are summarized in Table S4. Based on both statistical indicators, the VFT equation provides the best statistical fit to the data for Ge_5Se_{95}. The MYEGA equation yields the best fit for the $Ge_{10}Se_{90}$ viscosity data, while the remaining two studied compositions are most accurately described by the KSZ model. This trend is consistent with previous findings for pure selenium and for materials with high selenium content[31]. The reliability of the viscosity data obtained via PAMFT was validated using energy-dispersive X-ray spectroscopy (EDS). To verify that no compositional changes occurred during the filling of the capillary, the filled capillaries were sectioned perpendicularly at several locations (typically the beginning, middle, and end of the filled segment), and EDS measurements were performed at these positions. The compositional variations did not exceed ±1 at. %, which is slightly higher than the ±0.5 at. % variation observed in the starting fibers. This increased variation is attributed to the inherent difficulty of EDS measurements carried out on very small regions (the inner diameters of the filling capillaries ranged from 3.46 to 5.8 μm). The average compositions obtained from all measured points were 5.0 ± 0.4, 9.8 ± 0.3, 14.8 ± 0.1, and 20.1 ± 0.5 at. % of Ge, respectively, confirming that the sample compositions remained effectively unchanged during the PAMFT process.

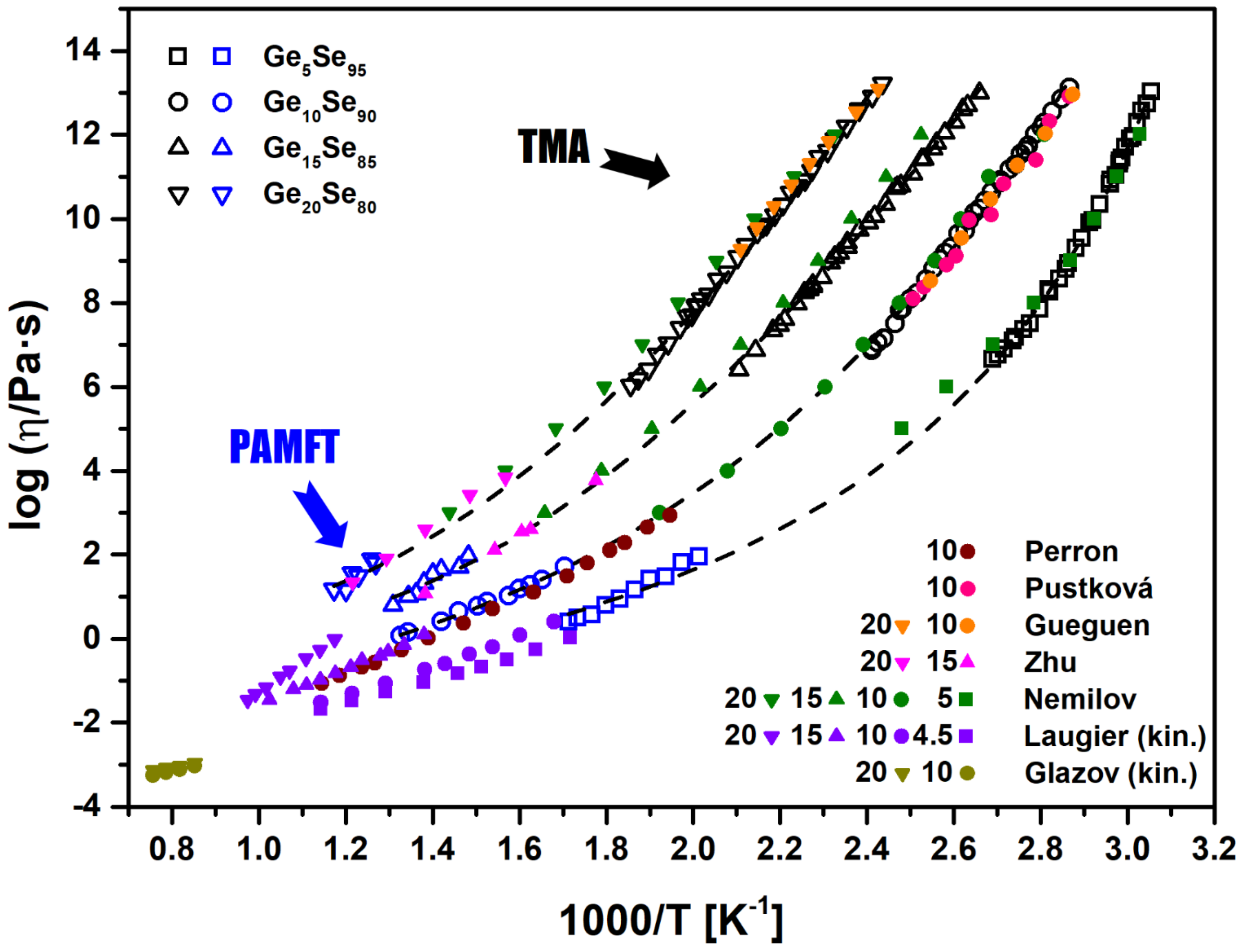

Figure S1. Temperature dependencies of viscosities for Ge_xSe_{100-x} glass-formers. Experimental data obtained in this study are shown alongside literature values reported by Perron et al.[17], Pustková et al.[18], Gueguen et al.[19], Zhu et al.[20], Nemilov[21], Laugier et al.[13], and Glazov and Situlina[14]. Specific germanium contents are indicated near the corresponding data points. Note that the latter two studies employed the oscillating cup method to determine kinematic viscosities. The dashed lines represent the fits to the data obtained in this study: the Vogel–Fulcher–Tammann (VFT) equation was applied to the Ge_5Se_{95} composition, the Mauro–Yue–Ellison–Gupta–Allan (MYEGA) equation to $Ge_{10}Se_{90}$, and the Krausser–Samwer–Zaccone (KSZ) equation to the remaining two compositions. The selection of the displayed fits was based solely on their statistical performance parameters (Table S4).

Table S2. Viscosities of Ge-Se glass and undercooled melts (TMA methods)

$Ge_{05}Se_{95}$		$Ge_{10}Se_{90}$		$Ge_{15}Se_{85}$		$Ge_{20}Se_{80}$	
T [K]	log(η/Pa·s)	T [K]	log(η/Pa·s)	T [K]	log(η/Pa·s)	T [K]	log(η/Pa·s)
327.4	13.02	348.8	13.12	376.1	12.99•	410.6	13.22
328.4	12.74	351.3	12.85	380.0	12.70•	414.6	12.91
329.9	12.57	353.8	12.55	381.7	12.60•	419.9	12.60•
330.9	12.30	356.3	12.28	384.3	12.30•	419.9	12.59•
331.9	11.95	356.9	12.17•	387.7	12.04•	425.0	12.19•
332.6	11.91•	358.8	12.02	390.7	11.80•	429.9	11.90•
333.9	11.70•	360.8	11.74	391.9	11.66•	433.1	11.61
334.9	11.45	361.8	11.63	395.0	11.42•	436.9	11.48•
335.3	11.38	363.2	11.54	396.0	11.41•	440.2	11.15
335.4	11.30	364.7	11.29•	399.2	11.06•	443.1	10.93
336.3	11.04	366.6	11.18	403.7	10.79•	446.2	10.78
337.7	10.93•	369.2	10.86•	405.0	10.75•	449.7	10.62
337.8	10.83	369.3	10.92	405.7	10.72	453.2	10.34
340.5	10.34	372.2	10.64	409.5	10.35•	457.2	10.09
342.5	9.96	374.2	10.43	413.7	10.06	461.3	9.86
343.4	9.90	376.0	10.24	415.9	9.91	463.1	9.82
345.3	9.54	377.7	10.14	419.9	9.73	465.8	9.67
347.1	9.30	379.3	9.97	424.7	9.44	471.4	9.38
349.2	8.93	380.7	9.72	424.7	9.32	475.8	9.09
349.8	8.79	383.1	9.65	427.9	9.18	481.6	8.72
351.8	8.57	385.5	9.32•	430.1	9.09	486.4	8.56
354.4	8.26	387.7	9.20	431.8	8.96	491.3	8.19
354.8	8.31	389.2	9.03	435.3	8.60	495.4	8.08=
357.4	7.85	391.9	8.81	439.6	8.43	497.9	7.95
360.2	7.51=	394.4	8.55	439.6	8.38	498.5	7.91
362.3	7.36=	397.4	8.23	441.1	8.31	500.7	7.71=
364.8	7.18=	400.2	8.08	443.3	8.24	503.4	7.67=
365.5	7.09=	403.0	7.85=	446.5	7.97	507.8	7.40=
368.3	6.90=	403.8	7.82	452.5	7.60	514.9	7.03=
370.1	6.75=	405.7	7.50=	455.0	7.45=	521.6	6.76=
371.8	6.65=	410.2	7.15=	458.0	7.34	527.1	6.41=
		412.3	7.05=	466.7	6.86=	533.8	6.19=
		414.6	6.86=	475.2	6.40=	538.9	6.03=
		414.6	6.93=				

Experimental points are marked according to a method used for their determination: parallel-plate method=, penetration with hemispherical indenter•, penetration with cylindrical indenter (without symbol). The accuracy of temperature measurement is ± 0.5 °C, and the accuracy of viscosity measurement is ± 0.1 log units[1].

Table S3. Viscosities of Ge-Se melts (PAMFT method)

$Ge_{05}Se_{95}$		$Ge_{10}Se_{90}$		$Ge_{15}Se_{85}$		$Ge_{20}Se_{80}$	
T [K]	log(η/Pa·s)	T [K]	log(η/Pa·s)	T [K]	log(η/Pa·s)	T [K]	log(η/Pa·s)
496.6	1.95	586.7	1.72	674.9	1.97	788.2	1.80
506.5	1.81	605.5	1.41	684.8	1.70	793.7	1.89
516.4	1.47	615.4	1.27	704.6	1.65	813.5	1.49
526.3	1.42	625.3	1.19	714.5	1.54	823.4	1.56
536.2	1.16	635.2	1.01	724.4	1.33	833.4	1.15
546.1	0.94	655.4	0.88	734.3	1.07	853.2	1.17
556.0	0.79	665.0	0.78	744.2	1.01		
565.9	0.57	684.8	0.65	764.1	0.80		
576.9	0.50	704.6	0.41				
583.8	0.40	744.3	0.16				
		754.2	0.07				

Table S4. Parameters of VFT, MYEGA and KSZ fits through viscosity data published in this work

Parameters	$Ge_{05}Se_{95}$			$Ge_{10}Se_{90}$		
	VFT	MYEGA	KSZ	VFT	MYEGA	KSZ
log(η_0/Pa·s)	-2.35 ± 0.09	-0.26 ± 0.10	0.68 ± 0.09	-3.89 ± 0.13	-2.17 ± 0.12	-0.07 ± 0.06
m	73.4 ± 0.9	67.7 ± 1.1	64.3 ± 1.4	45.1 ± 0.7	43.7 ± 0.5	41.6 ± 0.5
T_{12} [K]	332.0 ± 0.1	331.8 ± 0.2	331.7 ± 0.3	358.9 ± 0.3	358.9 ± 0.2	358.8 ± 0.3
R^2	0.99933	0.99861	0.99751	0.99903	0.99936	0.99931
AIC	-176.9	-146.8	-123.0	-175.9	-194.3	-191.3
Parameters	$Ge_{15}Se_{85}$			$Ge_{20}Se_{80}$		
	VFT	MYEGA	KSZ	VFT	MYEGA	KSZ
log(η_0/Pa·s)	-4.67 ± 0.16	-3.41 ± 0.18	-0.21 ± 0.09	-4.10 ± 0.22	-2.89 ± 0.24	0.14 ± 0.10
m	34.8 ± 0.5	34.2 ± 0.4	33.0 ± 0.4	33.6 ± 0.6	33.1 ± 0.5	31.9 ± 0.4
T_{12} [K]	388.4 ± 0.3	388.4 ± 0.3	388.3 ± 0.3	428.0 ± 0.5	428.1 ± 0.5	428.2 ± 0.4
R^2	0.99916	0.99927	0.99934	0.99803	0.99835	0.99885
AIC	-175.9	-181.5	-185.9	-138.8	-145.7	-159.9

References

(1) Koštál, P.; Hofírek, T.; Málek, J. Viscosity measurement by thermomechanical analyzer. *J. Non-Cryst. Solids* **2018**, *480*, 118-122. DOI: 10.1016/j.jnoncrysol.2017.05.027.

(2) Cox, S. M. A Method of Viscosity Measurement in the Region 10^8 Poises. *J. Sci. Instrum.* **1943**, *20*, 113-114.

(3) Exnar, P.; Hrubá, M.; Uhlíř, J.; Voldán, J. Experience with the Penetration Viscosimeter. *Silikáty* **1980**, *24*, 169-179.

(4) Douglas, R. W.; Armstrong, W. L.; Edward, J. P.; Hall, D. A penetration viscometer. *Glass Technol.* **1965**, *6*, 52-55.

(5) Yang, F. Q.; Li, J. C. M. Newtonian viscosity measured by impression test. *J. Non-Cryst. Solids* **1997**, *212*, 126-135.

(6) Yang, F. Q.; Li, J. C. M. Viscosity of selenium measured by impression test. *J. Non-Cryst. Solids* **1997**, *212*, 136-142.

(7) Fontana, E. H. A versatile parallel-plate viscometer for glass viscosity measurements to 1000 °C *Ceram. B.* **1970**, *49*, 594-597.

(8) International, A. *Standard Test Method for Linear Thermal Expansion of Solid Materials by Thermomechanical Analysis*; 2014.

(9) International, A. *Standard Test Method for Length Change Calibration of Thermomechanical Analyzers*; 2018.

(10) International, A. *Standard Test Method for Measurement of Viscosity of Glass Between 10^4 Pa·s and 10^8 Pa·s by Viscous Compression of a Solid Right Cylinder*; 2002.

(11) Koštál, P.; Včeláková, M.; Málek, J. Viscosity and heat capacity of As_2Se_3 connected via Adam-Gibbs model. *J. Am. Ceram. Soc.* **2024**, *107*, 844-858. DOI: 10.1111/jace.19491.

(12) Barták, J.; Koštál, P.; Valdes, D.; Málek, J.; Wieduwilt, T.; Kobelke, J.; Schmidt, M. A. Analysis of viscosity data in As2Se3, Se and Se95Te5 chalcogenide melts using the pressure assisted melt filling technique. *J. Non-Cryst. Solids* **2019**, *511*, 100-108. DOI: 10.1016/j.jnoncrysol.2019.01.037.

(13) Laugier, A.; Chaussemy, G.; Fornazero, J. Viscosity of Glass-Forming Ge-Se Liquid Solutions. *J. Non-Cryst. Solids* **1977**, *23*, 419-429.

(14) Glazov, V. M.; Situlina, O. V. Physicochemical analysis of Group IV element-selenium binary liquid systems. *Dokl. Akad. Nauk SSSR* **1969**, *187*, 799-802.

(15) Avetikyan, G. B.; Baidakov, L. A. Temperature dependence of the density of selenium-enriched glasses of the system Ge-Se and their thermal expansion. *Izv. Akad. Nauk SSSR Neorg. Mater.* **1972**, *8*, 1489-1490.

(16) Ruska, J.; Thurn, H. Change of short-range order with temperature and composition in liquid GexSe1-x as shown by density-measurements. *J. Non-Cryst. Solids* **1976**, *22*, 277-290. DOI: 10.1016/0022-3093(76)90059-4.

(17) Perron, J. C.; Rabit, J.; Rialland, J. F. Impurity Dependence of the Viscosity of Liquid Selenium. *Philos. Mag. B* **1982**, *46*, 321-330. DOI: 10.1080/13642818208246443.

(18) Pustkova, P.; Shanelova, J.; Malek, J.; Cicmanec, P. Relaxation behavior of selenium based glasses. *J. Therm. Anal. Calorim.* **2005**, *80*, 643-647. DOI: 10.1007/s10973-005-0707-5.

(19) Gueguen, Y.; Rouxel, T.; Gadaud, P.; Bernard, C.; Keryvin, V.; Sangleboeuf, J. C. High-temperature elasticity and viscosity of GexSe1-x glasses in the transition range. *Phys. Rev. B* **2011**, *84*, 064201. DOI: 10.1103/Physrevb.84.064201.

(20) Zhu, W.; Marple, M. A. T.; Lockhart, M. J.; Aitken, B. G.; Sen, S. An experimental critique on the existence of fragile-to-strong transition in glass-forming liquids. *J. Non-Cryst. Solids* **2018**, *495*, 102-106. DOI: 10.1016/j.jnoncrysol.2018.05.009.

(21) Nemilov, S. V. Viscosity and Structure of Se-Ge Glasses (russ.). *J. Appl. Chem.-USSR+* **1964**, *37*, 1020-1024.

(22) Webber, P. J.; Savage, J. A. Some physical properties of Ge-As-Se infrared optical glasses. *J. Non-Cryst. Solids* **1976**, *20*, 271-283.

(23) Senapati, U.; Varshneya, A. K. Viscosity of chalcogenide glass-forming liquids: An anomaly in the 'strong' and 'fragile' classification. *J. Non-Cryst. Solids* **1996**, *197*, 210-218.

(24) Meyer, O. E. Ein Verfahren zur Bestimmung der inneren Reibung von Flüssigkeiten. *Ann. Phys. Chem.* **1891**, *5*, 1-14.

(25) Shvidkovskii, E. G. *Some problems associated with the viscosity of molten metals*; 1955.

(26) Vogel, H. Das Temperatur-abhängigkeitsgesetz der Viskosität von Flüssigkeiten. *Phys. Z.* **1921**, *22*, 645-646.

(27) Fulcher, G. S. Analysis of recent measurements of the viscosity of glasses. *J. Am. Ceram. Soc.* **1925**, *8*, 339-355.

(28) Tammann, G.; Hesse, W. Die Abhängigkeit der Viscosität von der Temperatur bei unterkühlten Flüssigkeiten. *Z. Anorg. Allg. Chem.* **1926**, *156*, 245-247.

(29) Mauro, J. C.; Yue, Y. Z.; Ellison, A. J.; Gupta, P. K.; Allan, D. C. Viscosity of glass-forming liquids. *P. Natl. Acad. Sci. USA* **2009**, *106*, 19780–19784. DOI: 10.1073/pnas.0911705106.

(30) Krausser, J.; Samwer, K. H.; Zaccone, A. Interatomic repulsion softness directly controls the fragility of supercooled metallic melts. *P. Natl. Acad. Sci. USA* **2015**, *112*, 13762-13767. DOI: 10.1073/pnas.1503741112.

(31) Koštál, P.; Shánělova, J.; Málek, J. Viscosity of chalcogenide glass-formers. *Int. Mater. Rev.* **2020**, *65*, 63-101. DOI: 10.1080/09506608.2018.1564545.
